# Prophylactic effect of scopolamine butylbromide, a competitive antagonist of muscarinic acetylcholine receptor, on irinotecan-related cholinergic syndrome

**DOI:** 10.1007/s00280-018-3736-z

**Published:** 2018-12-18

**Authors:** Hirotoshi Iihara, Hironori Fujii, Chiaki Yoshimi, Ryo Kobayashi, Nobuhisa Matsuhashi, Takao Takahashi, Kazuhiro Yoshida, Akio Suzuki

**Affiliations:** 1 Department of Pharmacy, Gifu University Hospital, 1-1 Yanagido, Gifu, 501-1194 Japan; 2 Department of Surgical Oncology, Gifu University Graduate School of Medicine, Gifu, 501-1193 Japan

**Keywords:** Cholinergic syndrome, Irinotecan, Scopolamine butylbromide

## Abstract

**Background/aim:**

Cholinergic syndrome frequently occurs within the first 24 h after irinotecan injection. We evaluated the prophylactic effect of scopolamine butylbromide on irinotecan-related cholinergic syndrome.

**Patients and methods:**

Fifty-nine patients who received irinotecan-based regimens at our outpatient chemotherapy clinic between April 2013 and May 2014 were enrolled. Patients who developed irinotecan-related cholinergic syndrome were prophylactically administered scopolamine butylbromide at the next scheduled treatment. Risk factors for irinotecan-related cholinergic syndrome were determined using logistic regression analysis.

**Results:**

Irinotecan-related cholinergic syndrome occurred in 50.8% of patients. Scopolamine butylbromide administration significantly reduced the incidence to 3.4% (*P* < 0.01). The irinotecan dose (≥ 150 mg/m^2^) was the only risk factor associated with irinotecan-related cholinergic syndrome. The incidence of cholinergic syndrome in patients with this risk factor was 75%.

**Conclusion:**

Scopolamine butylbromide was effective in preventing irinotecan-related cholinergic syndrome. It is recommended for patients receiving ≥ 150 mg/m^2^ irinotecan who may develop cholinergic syndrome at high frequency.

## Introduction

Irinotecan, a topoisomerase I inhibitor, is a semisynthetic derivative of the plant alkaloid camptothecin which is used to treat a variety of tumors, including those associated with ovarian cancer, small cell lung cancer, cervical cancer, colon cancer and rectal cancer [1]. Irinotecan is a prodrug, and its active metabolite 7-ethyl-10-hydroxycamptothecin (SN-38) has both antitumor activities and toxicities [2, 3]. SN-38 is inactivated into SN-38 glucuronide (SN-38G) mainly by UDP-glucuronyltransferase 1A1 (UGT1A1) [2, 3]. Genetic polymorphisms of UGT1A1, such as wild-type allele (*1/*1), homozygous mutations (*28/*28, *6/*6 and *28/*6) and heterozygous mutations (*28/*1 and *6/*1), affect the glucuronidation activity of UGT1A1, and heterozygous and homozygous mutations lead to a lower rate of inactivation of SN-38 than the wild-type allele [4]. Genetic polymorphisms of UGT1A1 show ethnic differences, in which the allele frequency of UGT1A1*28 is lower in Asians than in Caucasians, while the frequency of UGT1A1*6 is less common in Caucasians compared to Asians [5]. Moreover, serious hematological toxicity is associated with UGT1A1*6 allele in Asians and are also associated with double heterozygosity (UGT1A1*6/*28) [6, 7]. Thus, genetic polymorphisms of UGT1A1 genes, including UGT1A1*6/*6, *28/*28 and *6/*28, are associated with the incidence of serious side effects in Asians unlike Caucasians [7, 8].

Clinical studies have shown that patients who receive irinotecan often experience acute adverse events, such as bradycardia, hypotension, hypersalivation, abdominal cramps, acute diarrhea, diaphoresis and other symptoms that are characteristic of cholinergic syndrome [9–11]. These symptoms are characterized by their occurrence during or shortly after administration of irinotecan and their amelioration within a few hours of completing the irinotecan injection [11]. The pathophysiological mechanisms underlying irinotecan-induced cholinergic syndrome remain to be clarified. Dodds and Rivory [12] demonstrated that irinotecan is a potent inhibitor of acetylcholinesterase at clinically relevant concentrations and revealed its mechanism of inhibition as being instantly reversible and apparently non-competitive. On the other hand, Blandizzi et al. [13] reported in an in vivo study that cholinergic syndrome does not arise due to the inhibition of acetylcholinesterase by irinotecan and SN-38. Instead, they demonstrated that irinotecan activated various nerve fibers and induced vagal reflexes at peripheral sites to trigger a cholinergic response. It is therefore to note that the management of acute diarrhea induced by irinotecan differs from delayed diarrhea occurring more than 24 h after irinotecan administration which is induced by exposure of intestinal epithelia to the released SN-38 [14].

Several reports have shown that the symptoms associated with irinotecan injection can be prevented or ameliorated by premedication with anticholinergic drugs such as atropine, scopolamine and scopolamine butylbromide [15–17]. Scopolamine butylbromide, unlike atropine and scopolamine which are tertiary amines, is a quaternary ammonium derivative and has little effect on the central nervous system because of passing through the blood–brain barrier [18–20]. However, the prophylactic effect of anticholinergic drugs on irinotecan-related cholinergic syndrome is unclear in Japanese patients receiving irinotecan. In addition, the prophylactic effect of anticholinergic drugs on irinotecan-related cholinergic syndrome in patients who develop irinotecan-related cholinergic syndrome while receiving irinotecan-based regimens has not been studied.

We examined the prophylactic effect of scopolamine butylbromide on irinotecan-related cholinergic syndrome at the next scheduled treatment with irinotecan in Japanese patients who developed this cholinergic syndrome. Moreover, we identified the risk factors associated with the development of irinotecan-related cholinergic syndrome.

## Patients and methods

### Study design and patients

Irinotecan-induced hyperhidrosis, abdominal pain, rhinitis and acute diarrhea that developed within 24 h after irinotecan administration were defined as cholinergic syndrome. Patients who received irinotecan-based regimens in our outpatient chemotherapy clinic between April 2013 and May 2014 were enrolled. Among these patients, those who developed cholinergic syndrome were prophylactically administered scopolamine butylbromide at the next scheduled treatment with irinotecan.

Incidence of cholinergic syndrome during or after administration of irinotecan was monitored by nurses, pharmacists and physicians. All patients were provided with a daily checklist to record adverse events, at their first visit to the outpatient chemotherapy clinic. The medical staffs asked the occurrence of cholinergic syndrome in an interview for all patients who visited the next cycle, and recorded it on an electronic medical chart. The symptoms monitored included hyperhidrosis, abdominal pain, diarrhea and rhinitis. The severity of cholinergic syndrome was graded according to the Common Terminology Criteria for Adverse Events (CTCAE, National Cancer Institute, MD, USA) version 4.0. Rhinitis was evaluated based on allergic rhinitis in CTCAE version 4.0.

### Prophylactic administration of scopolamine butylbromide

A 20-mg scopolamine butylbromide injection was mixed with normal saline or dextrose 5% in water used to dissolve irinotecan, and the solution was infused intravenously over 90 min.

### Risk analysis for irinotecan-related cholinergic syndrome

Demographics of patients who received the irinotecan-based regimens were compared between those who did and did not develop cholinergic syndrome. A *P* value was calculated for each demographic item. Items for which the *P* value was ≤ 0.05 were subsequently tested using logistic regression analysis. Receiver operating characteristic (ROC) curves were used to determine the cutoff of the irinotecan dose for logistic regression analysis.

### Data analysis

Parametric analysis was conducted using a *t* test, while nonparametric analysis was performed using a Mann–Whitney *U* test, McNemar’s test or Chi squared test. Data were analyzed using SPSS version 22 (SPSS Inc., Chicago, IL, USA). *P* values less than 0.05 were considered statistically significant.

### Ethics statement

The present study was conducted according to the guidelines for human studies of the ethics committee of Gifu University Graduate School of Medicine and the Government of Japan, and was approved by the university’s institutional review board (Approval no. 26-153). In view of the retrospective nature of the study, informed consent from the subjects was not mandated.

## Results

### Patient demographics

Patient demographics are shown in Table 1. A total of 59 patients (39 men and 20 women) were enrolled in the present study. The mean age was 66 years (range 34–85 years). The most common cancer type was colorectal cancer (*n* = 39, 66.1%), followed by gastric cancer (*n* = 16, 27.1%) and lung cancer (*n* = 4, 6.8%). In contrast, the most common chemotherapy regimen was the irinotecan + 5-fluorouracil + folinic acid (FOLFIRI)-based regimen (*n* = 31, 52.5%), followed by irinotecan + tegafur/oteracil (IRIS)-based regimen (*n* = 12, 20.3%), irinotecan + cisplatin regimen (*n* = 9, 15.3%) and monotherapy regimen (*n* = 7, 11.9%).

**Table 1:** Patient demographics

Number of patients, *n* (male/female)	59 (39/20)
Age, mean (range)	66 (34–85)
Height (cm)^a^	160.2 ± 7.7
Weight (kg)^a^	55.9 ± 10.7
Body surface area (m^2^)^a^	1.57 ± 0.16
Body mass index (kg/m^2^)^a^	21.7 ± 3.6
Dose of irinotecan (mg/m^2^)^a^	117.5 ± 36.7
Cancer type, *n* (%)	
Colon	39 (66.1)
Gastric	16 (27.1)
Lung	4 (6.8)
Chemotherapy regimen, *n* (%)	
FOLFIRI-based	31 (52.5)
IRIS-based	12 (20.3)
CPT-11 + CDDP	9 (15.3)
Monotherapy	7 (11.9)

*FOLFIRI* 5-fluorouracil + folinic acid + irinotecan, *CPT-11* irinotecan, *CDDP* cisplatin, *IRIS* irinotecan + tegafur/gimeracil/oteracil

^a^Data indicate mean ± standard deviation

### Incidence of irinotecan-related cholinergic syndrome and the effect of scopolamine butylbromide

The overall incidence of irinotecan-related cholinergic syndrome was 50.8% (30/59). The most common symptom of irinotecan-related cholinergic syndrome was hyperhidrosis (*n* = 23, 76.7%; grade 1: *n* = 15; grade 2: *n* = 8), followed by abdominal pain (*n* = 10, 33.3%; grade 1: *n* = 8; grade 2: *n* = 2), rhinitis (*n* = 8, 26.7%; grade 1: *n* = 8) and diarrhea (*n* = 2, 6.7%, grade 1: *n* = 2) (Table 2).

**Table 2:** Type and severity of symptoms of irinotecan-related cholinergic syndrome observed in 30 patients during or after intravenous infusion of irinotecan

Symptom	Grade 1	Grade 2	Total (%)
Hyperhidrosis	15	8	23 (76.7)
Abdominal pain	8	2	10 (33.3)
Rhinitis	8	0	8 (26.7)
Diarrhea	2	0	2 (6.7)

**Fig. 1:**
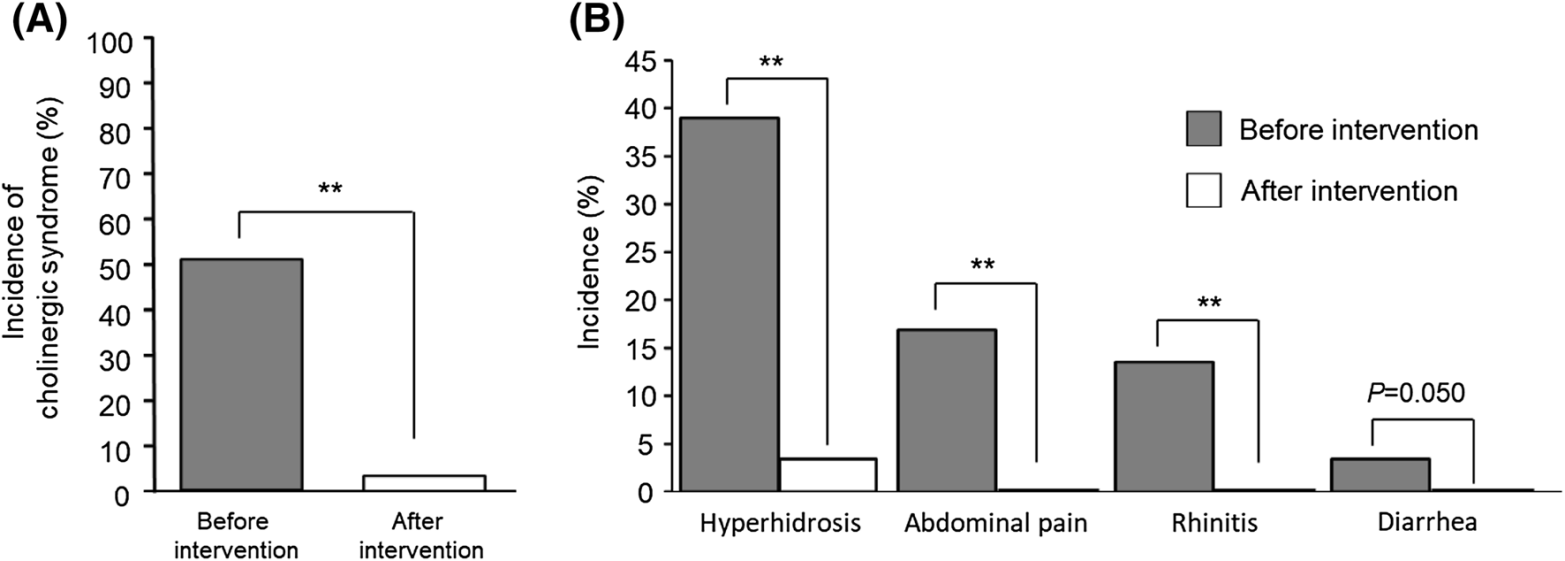
Overall incidence of irinotecan-related cholinergic syndrome (**a**) and incidence according to individual symptoms (**b**) before and after intervention with prophylactic administration of scopolamine butylbromide in patients who developed cholinergic syndrome. McNemar’s test was used to analyze the data. **P < 0.01

The 30 patients who developed irinotecan-related cholinergic syndrome were prophylactically treated with scopolamine butylbromide at the next scheduled treatment with irinotecan. As shown in Fig. 1a, the overall incidence of irinotecan-related cholinergic syndrome was significantly reduced by the prophylactic administration of scopolamine butylbromide (50.8% vs. 3.4%, *P* < 0.01). Moreover, the following symptoms of irinotecan-related cholinergic syndrome were significantly or almost completely reduced by treatment with scopolamine butylbromide: hyperhidrosis (30.5% vs. 3.4%, *P* < 0.01), abdominal pain (16.9% vs. 0%, *P* < 0.01), rhinitis (11.9% vs. 0%, *P* < 0.01) and diarrhea (3.4% vs. 0%, *P* = 0.05) (Fig. 1b).

### Risk factors associated with the incidence of irinotecan-related cholinergic syndrome

To determine the risk factors associated with irinotecan-related cholinergic syndrome, the demographics of patients were compared between patients who did and did not develop irinotecan-related cholinergic syndrome. As shown in Table 3, the dose of irinotecan (135.2 ± 25.8 mg/m^2^ vs. 99.3 ± 37.7 mg/m^2^, *P* < 0.001) and incidence of colon cancer (83.3% vs. 48.3%, *P* = 0.010) were significantly different between the two groups.

**Table 3:** Comparison of the characteristics of patients who did and did not develop irinotecan-related cholinergic syndrome

Characteristic	Cholinergic syndrome		*P* value
	With (*n* = 30)	Without (*n* = 29)	
Gender, *n* (male/female)	18/12	21/8	0.464^a^
Age, mean (range)	67.5 (51–85)	65.3 (34–85)	0.660^b^
Height (cm)^d^	159.0 ± 8.0	161.4 ± 7.2	0.233^c^
Weight (kg)^d^	54.8 ± 9.2	57.1 ± 12.1	0.411^c^
Body surface area (m^2^)^d^	1.55 ± 0.10	1.59 ± 0.18	0.330^c^
Body mass index (kg/m^2^)^d^	21.6 ± 3.2	21.8 ± 3.9	0.824^c^
Dose of irinotecan (mg/m^2^)^d^	135.2 ± 25.8	99.3 ± 37.7	< 0.001^c^
Cancer type, *n* (%)			
Colon	25 (83.3)	14 (48.3)	0.010^a^
Gastric	3 (10.0)	13 (44.8)	0.007^a^
Lung	2 (6.7)	2 (6.9)	1.000^a^
Chemotherapy regimen, *n* (%)			
FOLFIRI-based	19 (63.3)	12 (41.4)	0.091^a^
IRIS-based	6 (20.0)	6 (20.7)	0.800^a^
CPT-11 + CDDP	1 (3.3)	8 (27.6)	0.026^a^
Monotherapy	4 (13.3)	3 (10.3)	1.000^a^

*FOLFIRI* 5-fluorouracil + folinic acid + irinotecan, *CPT-11* irinotecan, *CDDP* cisplatin, *IRIS* irinotecan + tegafur/gimeracil/oteracil

^a^Fisher’s exact probability test

^b^Mann–Whitney *U* test

^c^*t* test

^d^Data indicate mean ± standard deviation

**Fig. 2:**
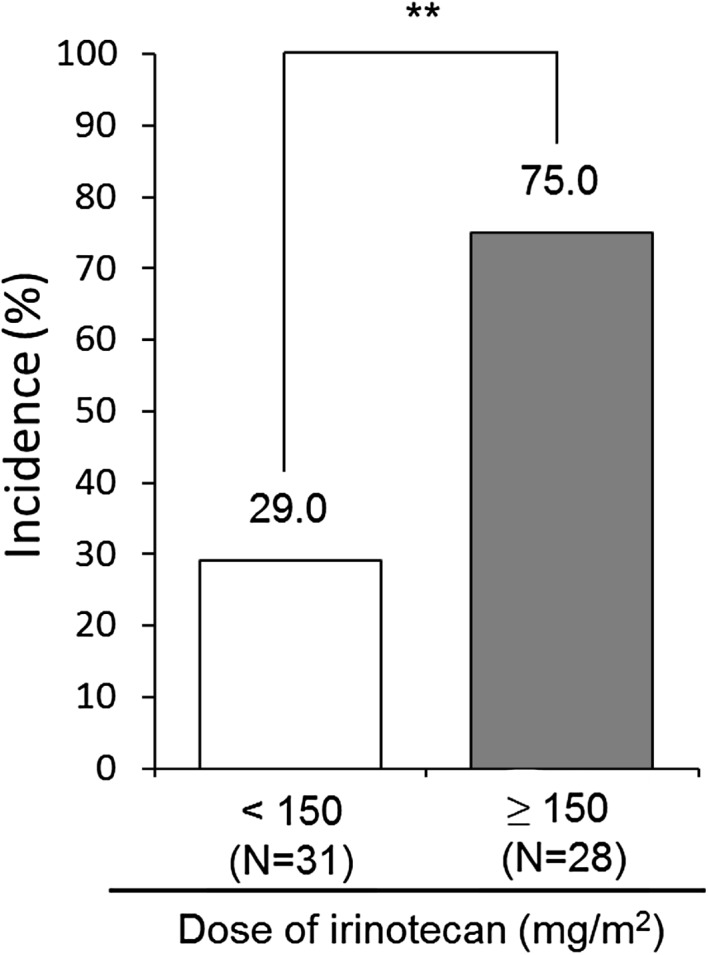
Comparison of the incidence of irinotecan-related cholinergic syndrome between patients who did and did not receive irinotecan at 150 mg/m^2^ or greater. Chi squared test was used to analyze the data. **P < 0.01

Univariate logistic regression analysis revealed that the dose of irinotecan (≥ 150 mg/m^2^) [hazard ratio (HR) 7.333, 95% confidence interval (CI) 2.311–23.267, *P* = 0.001] and incidence of colon cancer (HR 5.357, 95% CI 1.605–17.879, *P* = 0.006) were significant risk factors for irinotecan-related cholinergic syndrome. Multivariate logistic regression analysis showed that only the dose of irinotecan (≥ 150 mg/m^2^) (HR 5.042, 95% CI 1.455–17.479, *P* = 0.011) was a significant risk factor (Table 4). Moreover, the incidence of cholinergic syndrome in patients who received irinotecan at 150 mg/m^2^ or greater was significantly higher than that in patients who received irinotecan at less than 150 mg/m^2^ (75.0% vs. 29.0%, *P* < 0.001) (Fig. 2).

**Table 4:** Univariate and multivariate logistic regression analyses for factors associated with the incidence of irinotecan-related cholinergic syndrome

Factor	Univariate logistic regression analysis			Multivariate logistic regression analysis		
	HR	95% CI	*P* value	HR	95% CI	*P* value
Dose of CPT-11 (≥ 150 mg/m^2^)	7.333	2.311–23.267	0.001	5.042	1.455–17.479	0.011
Colon cancer	5.357	1.605–17.879	0.006	2.770	0.723–10.615	0.137

*HR* hazard ratio, *95% CI* 95% confidence interval, *CPT-11* irinotecan, *CDDP* cisplatin

## Discussion

We found that 50.8% (30/59) of patients who received irinotecan-based regimens in our outpatient chemotherapy clinic developed irinotecan-related cholinergic syndrome, with varying rates of hyperhidrosis (76.7%), abdominal pain (33.3%), rhinitis (26.7%) and diarrhea (6.7%). A wide variation in the incidence of overall irinotecan-related cholinergic syndrome, ranging from 31.3 to 83.0%, has been reported in patients receiving chemotherapies including irinotecan [11, 21, 22]. Such a difference in the incidence of this cholinergic syndrome may be attributable to differences in the dose of irinotecan. In our study, patients were treated with irinotecan (55–152 mg/m^2^) administered by intravenous infusion for 90 min. In contrast, Pitot et al. conducted a Phase I study in 34 patients with advanced refractory solid malignancies who were treated with irinotecan (240–340 mg/m^2^) administered by intravenous infusion for 90 min. They reported that the incidence of cholinergic symptoms ranged from 33% for patients who received 240 mg/m^2^ doses to 83% for patients treated with a starting dose of 340 mg/m^2^ [21]. Further, Kanbayashi et al. [22] conducted a retrospective study in 150 cancer outpatients treated with 34.7–180.0 mg/m^2^ irinotecan, and reported that cholinergic syndrome, graded according to their original criteria, occurred in 31.3% of patients.

In this study, patients who experienced irinotecan-related cholinergic syndrome were prophylactically administered scopolamine butylbromide at the next scheduled treatment with irinotecan. Prophylactic administration of scopolamine butylbromide significantly reduced the overall incidence of irinotecan-related cholinergic syndrome (50.8% vs. 3.4%, *P* < 0.01). Moreover, all symptoms of cholinergic syndrome including hyperhidrosis, abdominal pain, rhinitis, and diarrhea were also significantly reduced by this intervention. Scopolamine butylbromide, a competitive antagonist of muscarinic acetylcholine receptors, is a quaternary ammonium derivative, and does not pass through the blood–brain barrier. As a consequence, scopolamine butylbromide has little central effects, such as sedation, confusion or paradoxical excitation [18]. In fact, no central nervous system adverse events associated with scopolamine butylbromide were observed in the present study (data not shown).

Two reports showed the prophylactic effect of atropine and scopolamine on irinotecan-related cholinergic syndrome [15, 16]. Cheng et al. reported a retrospective, nonrandomized, cohort study in 80 cancer patients who were pre-treated with atropine diphenoxylate or hyoscyamine before receiving irinotecan. The overall incidence of cholinergic syndrome was not significantly different between the atropine–diphenoxylate (8.2%) and hyoscyamine (9.0%) groups (*P* = 0.760) [15]. Yumuk et al. conducted a retrospective analysis in 66 metastatic colorectal cancer patients who received 85 mg/m^2^ irinotecan once a week or 350 mg/m^2^ irinotecan every 3 weeks. All patients were administrated atropine sulfate subcutaneously before irinotecan infusion, and no cholinergic symptoms, specifically early diarrhea, were observed [16]. In both reports, anticholinergic drugs were prophylactically administered to all patients who received irinotecan, unlike in the present study. Additionally, atropine and hyoscyamine, which are tertiary amines, pass through the blood–brain barrier and can cause central effects, such as sedation, confusion, or paradoxical excitation, especially in the elderly [19, 20]. In contrast, in the third report, Zampa et al. [17] investigated the prophylactic effect of scopolamine butylbromide on irinotecan-related cholinergic syndrome in 13 patients who were administered scopolamine butylbromide 30 min before irinotecan. Scopolamine butylbromide was administered to 2 patients who showed evidence of cholinergic syndrome symptoms and subsequently to all other patients to prevent these symptoms. No further patients showed cholinergic syndrome symptoms. However, it is important to note that the sample size of this study was very small.

Several studies have reported that the development of irinotecan-related cholinergic syndrome is dose dependent [9, 21, 22]. Our multivariate logistic regression analysis showed that an irinotecan dose of 150 mg/m^2^ or greater was the only risk factor for the development of irinotecan-related cholinergic syndrome, as determined using ROC curves. Recently, Kanbayashi et al. [22] reported that irinotecan dose (≥ 175 mg), in addition to female sex, was a significant risk factor for developing irinotecan-related cholinergic syndrome, which is mostly consistent with our present finding. We found that 75% of patients who received irinotecan doses of 150 mg/m^2^ or greater developed irinotecan-related cholinergic syndrome. Therefore, prophylactic administration of scopolamine butylbromide is recommended for the treatment of irinotecan-related cholinergic syndrome in patients with this risk factor.

There were several limitations in the present study. First, this was a retrospective study; therefore, potentially relevant confounding factors may have been excluded. Second, the sample size was small and data were obtained from a single institution. Therefore, a larger scale, randomized control study is needed to confirm the prophylactic effect of scopolamine butylbromide against irinotecan-related cholinergic syndrome in patients receiving irinotecan-based regimens.

In conclusion, scopolamine butylbromide was effective in reducing the incidence of irinotecan-related cholinergic syndrome among patients receiving irinotecan-based regimens who developed cholinergic syndrome. Scopolamine butylbromide, unlike atropine and scopolamine, has no central effects. In addition, an irinotecan dose of 150 mg/m^2^ or greater was a risk factor for irinotecan-related cholinergic syndrome. Therefore, prophylactic administration of scopolamine butylbromide is recommended for patients receiving irinotecan doses ≥ 150 mg/m^2^ who develop irinotecan-related cholinergic syndrome.
